# Flame Retardancy and Excellent Electrical Insulation Performance of RTV Silicone Rubber

**DOI:** 10.3390/polym13172854

**Published:** 2021-08-25

**Authors:** Muhammad Tariq Nazir, Arslan Khalid, Imrana Kabir, Cheng Wang, Juan-Carlos Baena, Shakeel Akram, Muhammad Shoaib Bhutta, Ghulam Yasin, Bao Toan Phung, Guan Heng Yeoh

**Affiliations:** 1 School of Mechanical and Manufacturing Engineering, University of New South Wales, Sydney, NSW 2052, Australia; arslan.khalid@unsw.edu.au (A.K.); i.kabir@unsw.edu.au (I.K.); c.wang@unsw.edu.au (C.W.); juan.baenavargas@unsw.edu.au (J.-C.B.); g.yeoh@unsw.edu.au (G.H.Y.); 2 Institut d’Electronique et des Systèmes (IES), UMR 5214, CNRS, Université de Montpellier, 34095 Montpellier, France; shakeel.akram@ies.univ-montp2.fr; 3 Binjiang College, Nanjing University of Information Science & Technology, Wuxi 214105, China; shoaibbhutta@hotmail.com; 4 Institute for Advanced Study, Shenzhen University, Shenzhen 518060, China; yasin@szu.edu.cn; 5 School of Electrical Engineering and Telecommunications, University of New South Wales, Sydney, NSW 2052, Australia; toan.phung@unsw.edu.au

**Keywords:** silicone rubber, flame retardancy, combustibility, electrical insulation, dielectric response, dielectric breakdown

## Abstract

Room temperature vulcanized (RTV) silicone rubber filled with aluminum trihydrate (ATH) is substantially engaged in electrical outdoor insulation applications. The pristine silicone rubber is highly combustible. ATH filled silicone rubber offers excellent electrical insulation but lacks in providing adequate flame retardancy. This short communication reports the novel results on improved flame retardancy of pristine and ATH filled silicone rubber whilst retaining the electrical insulation properties to a great extent. Results suggest that the presence of only one percent of graphene nanoplatelets with ATH sharply reduces the heat release rate and rate of smoke release. A minor reduction in dielectric breakdown strength and volume resistivity is noticed. Furthermore, permittivity and dielectric loss at power frequency suggest that a marginal 1% concentration of nanoplatelet with ATH is an excellent approach to fabricate flame retardant silicone rubber with an acceptable electrical insulation level.

## 1. Introduction

Room temperature vulcanized (RTV) silicone rubber-based composites are widely used in the electrical industry and power system equipment for electrical insulation purposes [1,2,3]. The major attribute of RTV silicone rubber is its hydrophobic nature. Hence, it has been applied to conventional ceramic and glass insulation to help preventative maintenance activities and avoid flashovers on high voltage transmission lines [4]. One drawback of the pristine form of RTV silicone rubber is that it couldn’t offer the required thermal, mechanical, electrical tracking, and erosion resistance performance. Moreover, it is well known that due to its organic nature, silicone rubber tends to degrade in an outer environment and is inherently combustible [5,6]. The silicone rubber quickly burns with a high heat release and rate of release of smoke production.

Recently, numerous studies have been reported on the electrical insulation performance of RTV silicone rubber insulation. The available literature provides the electrical tracking and erosion studies of ATH, silica, alumina, AlN, BN, etc. [7,8,9,10]. Ilhan et al. [11] investigated the development of leakage current on the RTV coated porcelain insulators in a salt fog chamber. Two RTV coatings doped with alumina trihydrate (ATH) and ground silica were compared at the same additive levels. It was found that higher leakage current developed in ATH-filled coating compared with its counterpart led to arc flashover. Moreover, Lan et al. [12] reported the impact of environmental pollution settling on RTV coatings’ deterioration in the coal-ash polluted areas. The coatings were synthesized by incorporating ATH in RTV. The results suggested that erosion on the coating was extensive due to pollution.

As far as flame retardancy of silicone rubber is concerned, Imrana et al. [13] recently reported the improved fire retardancy of polydimethylsiloxane (PDMS) filled with multi-walled carbon nanotubes. The peak heat release rate, peak smoke production rate, total smoke release rate, carbon monoxide, and carbon dioxide production were measured 42%, 47%, 18%, 28%, and 47% less, respectively, in a PDMS/Surfactant/MWCNT–COOH than the pristine PDMS. Chen et al. [14] investigated the co-filled impact of aluminum phosphate and expandable graphite on the fire retardancy of PDMS and concluded that it could be an excellent approach to enhance the fire retardancy of PDMS. Moreover, Liu et al. [15] reported the smoke suppression and flame retardancy of silicone rubber filled with containing intumescent flame retardant and ferric hydroxide (FeOOH). Results suggested that FeOOH could impart excellent flame retardancy and thermal stability during the combustibility of silicone rubber.

It is well known that the bandwidth for the selection of flame retardants to be used in the electrical industry is very narrow since all conventional intumescent fire retardants are electrically conductive whilst present-day RTV coating technology filled with ATH, and silica particles impart good pollution flashover and electrical tracking resistance but lacks adequate flame retardancy to some extent. Therefore, a new concept of ATH assisted with pure graphene is explored in this work to achieve excellent flame retardancy with the superb electrical insulation characteristics of RTV silicone rubber.

## 2. Materials and Methods

**Figure 1:**
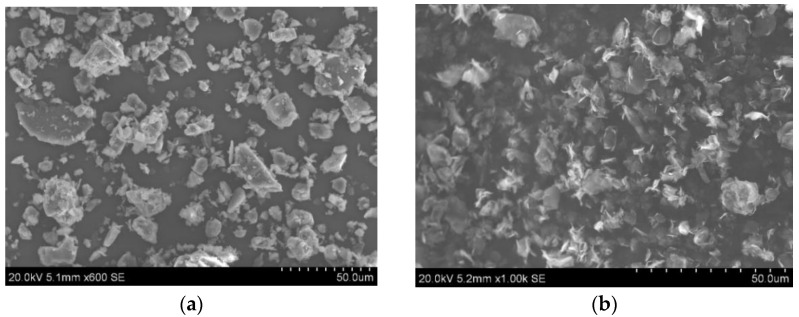
SEM images of (**a**) ATH and (**b**) graphene nanoplatelets.

RTV silicone rubber (trade name of RTV 615) was procured from DC product Melbourne. RTV 615 is a two-part platinum catalyst cured silicone rubber with a density of 1.01 g·cm^3^ and a viscosity of 4300 cps. ATH with a 5-micron size was supplied by Redox Pty Ltd., Sydney, Australia [16]. Graphene (PureGRAPH 5) is provided by First graphene Ltd. Australia with a size of 5 microns [17]. Figure 1 shows the SEM images of ATH, and graphene used in this study.

**Figure 2:**
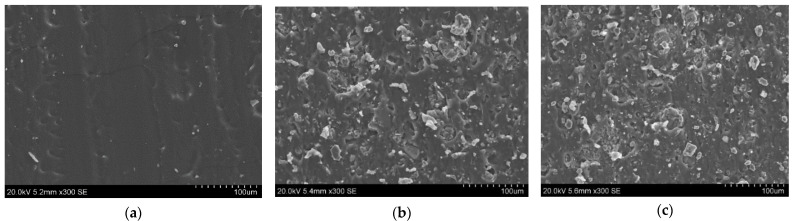
SEM images of (**a**) SR, (**b**) SRH and (**c**) SRHG.

Firstly, the required amount of part A of RTV 615 was taken and vacuumed in order to remove the trapped air in it. ATH and graphene nanoplatelets were kept in a laboratory overnight for drying purposes. The required amount of ATH and graphene nanoplatelets were mixed in part A of RTV 615 using a sharp blade mechanical mixer and the matrix was degassed. Subsequently, part B of RTV 615 was mixed in the cross-composite matrix by a ratio of 10:1. A detailed fabrication method is reported earlier in our previous article [18]. The composition of samples used in this study are given in Table 1. Figure 2 is showing the SEM images captured on the cross-section of the samples.

## 3. Results and Discussion

### 3.1. Combustibility and Release of Smoke

**Figure 3:**
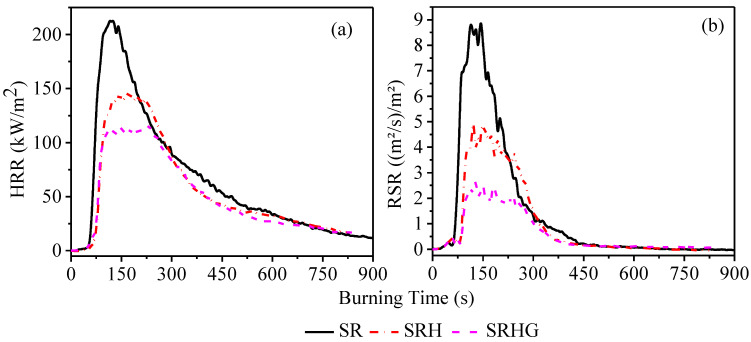
Combustibility of RTV samples (**a**) heat release rate (HRR) and (**b**) rate of release of smoke (RSR).

Cone calorimetry provides high-quality quantitative information about the combustibility and smoke release of any material. The FTT iCone Classic is coupled with pre-packed data acquisition hardware and Cone Calc software is used for experiments. Cone calorimeter parameters, such as heat release rate (HRR) and rate of smoke release (RSR) are thoroughly studied to explore the fire and smoke suppression of RTV coatings. Cone calorimeter experiments are conducted at a heat flux of 35 kW/m^2^. The HRR and RSR profiles of samples are depicted in Figure 3. The peak values of HRR and RSR in pristine SR samples are measured at 213 kW/m^2^ and 9 (m^2^/s)/m^2^, respectively. The addition of ATH imparts a significant positive impact on the combustibility of the SRH and both HRR and RSR parameters descend sharply. The most striking aspect of the results is seen in SRHG as shown in Figure 3. The presence of 1% of graphene nanoplatelets in SRHG sharply reduces the peak values of HRR and RSR to 116 kW/m^2^ and 2.5 (m^2^/s)/m^2^, respectively. These results suggest that ATH significantly contribute to flame retardancy of RTV whilst the substitution of the graphene nanoplatelets in solely ATH filled RTV renders a sharp reduction in HRR and RSR results. It is highly likely that the presence of graphene nanoplatelets substantially contributes to the formation of the carbonized protective layer during burning which acts as a strong barrier against combustibility, HRR, and RSR [19].

### 3.2. Limiting Oxygen Index (LOI)

The limiting oxygen index (LOI) is the minimum oxygen concentration that supports the combustion of polymers. LOI of RTV samples are measured using the FTT Oxygen Index apparatus in accordance with ASTM D 28663. The LOI of SR, SRH and SRHG are measured at 26%, 30% and 35%, respectively. A large increase in the LOI value of SRHG could be due to the presence of a marginal amount of graphene nanoplatelets. Interestingly, the LOI results are found to be consistent with the HRR and RSR findings.

### 3.3. Dielectric Breakdown Strength and Volume Resistivity (DC)

**Figure 4:**
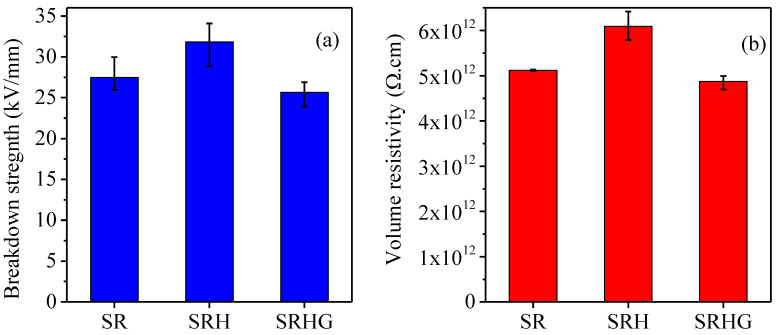
Results on (**a**) breakdown strength and (**b**) volume resistivity.

AC breakdown tests are performed as per the IEC60243-1 Standard. Spherical electrodes with a diameter of 25 mm are used in this work. The test sample are sandwiched between electrodes and the whole setup is immersed in transformer oil to avoid surface breakdown. Moreover, applied AC stress of 50 Hz is ramped up at a rate of 2 kV/s until breakdown occurs in the sample. Moreover, a Keithley 8009 resistivity test fixture is used for DC volume resistivity measurement of samples as per ASTM D257. A 40 V DC voltage is applied, and volume resistivity is measured concurrently over a period of 1 minute. The average breakdown strength and volume resistivity results are shown in Figure 4. The average breakdown strengths of SR, SRH and SRHG are measured at 27.47, 31.84 and 25.65 kV/mm for SR, SRH and SRHG, respectively. A similar trend is seen in the volume resistivity of the samples. The volume resistivities of SR, SRH, and SRHG are found at 5.12 × 10^12^, 6.09 × 10^12^ and 4.87 × 10^12^ Ω·cm, respectively. It is well known that graphene is a flame retardant but exhibits good electrical conductivity. From the results, it is evident that 1% of graphene nanoplatelets marginally reduced the electrical breakdown strength and volume resistivity but on the other side, it renders excellent improvement in fire retardancy. Hence, the synergy of optimized concentration of ATH/graphene could be the key for achieving good flame retardancy with desired electrical insulation of RTV.

### 3.4. Permittivity and Dielectric Loss

**Figure 5:**
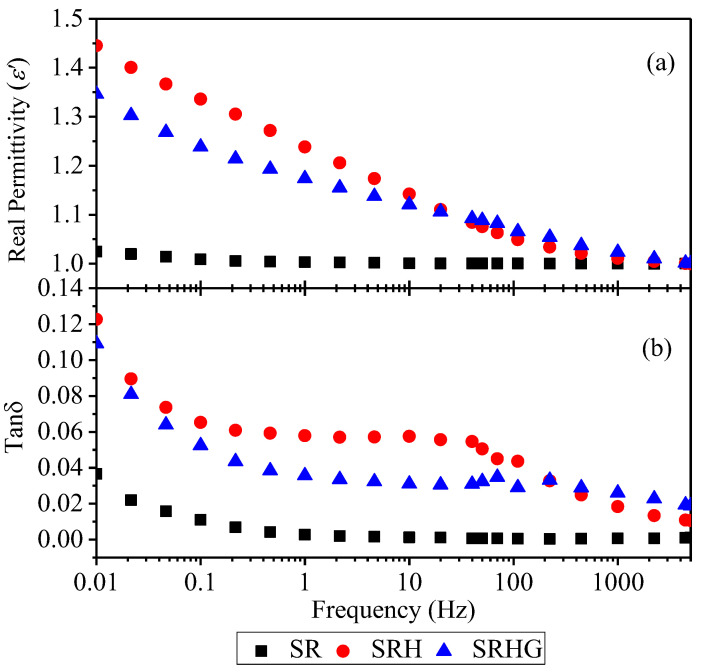
Dielectric response of samples (**a**) real permittivity and (**b**) dielectric loss tangent.

Dielectric response of RTV samples is measured through the OMICRON Dirana setup. The dielectric response is carried out using a sinusoidal voltage of 200 V peak amplitude over the frequency from 0.01 Hz to 5000 Hz. The dielectric response is found to solely depends on the introduction of ATH and graphene nanoplatelets in the RTV. The permittivity and dielectric loss tangent results are plotted as shown in Figure 5. It is observed that there is an increment in permittivity and dielectric loss values from higher to lower frequencies. This increment is found sharper in SRH and SRHG relative to SR as shown in Figure 5. This could be because of dominant dipole polarization at the lower frequencies whilst dipoles are more restricted at higher frequencies and not able to respond quickly. With the addition of ATH and graphene nanoplatelets, the permittivity and dielectric loss values are increased, and this could be because of interfacial polarization which is introduced by the particles. At a 50 Hz power frequency, the permittivity of SR, SRH and SRHG are measured at 1.00048, 1.07572 and 1.08819 whilst the dielectric loss are measures at 0.00065, 0.05053 and 0.03233, respectively. A good dielectric insulating material must offer low dielectric loss at the operating power frequency. From above, it can be concluded that the marginal 1wt% of graphene nanoplatelets can be a valuable addition to ATH filled RTV silicone rubber to enhance the fire retardancy with excellent dielectric properties.

## 4. Conclusions

The flame retardancy and electrical insulation performance of ATH filled silicone rubber assisted with graphene nanoplatelets is studied in this work. Results suggest that the addition of graphene nanoplatelets in the ATH filled silicone rubber substantially reduce the combustibility whilst maintain the electrical insulation properties to an acceptable level. It is found that the presence of 1% of graphene nanoplatelets in SRHG sharply reduces the peak values of HRR and RSR to 116 kW/m^2^ and 2.5 (m^2^/s)/m^2^, respectively. The LOI of SR, SRH and SRHG are measured at 26%, 30% and 35%, respectively. Moreover, it is concluded that the average breakdown strengths of SR, SRH and SRHG are measured at 27.47, 31.84 and 25.65 kV/mm for SR, SRH and SRHG, respectively. The volume resistivities of SR, SRH and SRHG are measured at 5.12 × 10^12^, 6.09 × 10^12^ and 4.87 × 10^12^ Ω·cm. Furthermore, the permittivity of SR, SRH and SRHG are measured at 1.00048, 1.07572 and 1.08819 whilst the dielectric loss are measures at 0.00065, 0.05053 and 0.03233, respectively. It is concluded that 1% of graphene nanoplatelets addition in solely ATH filled silicone rubber can help to enhance flame retardancy by retaining an optimal level of electrical insulation.

## Figures and Tables

**Table 1:** Formulations used in this work.

Composition	RTV 615	ATH	Graphene Nanoplatelets	Acronym
Pristine RTV silicone rubber	100%	0	0	SR
RTV Silicone rubber/ATH	70%	30%	0	SRH
RTV Silicone rubber/ATH/Graphene nanoplatelets	69%	30%	1%	SRHG

## Data Availability

Not applicable.
